# Solvent-Free Synthesis of Multifunctional Block Copolymer and Formation of DNA and Drug Nanocarriers

**DOI:** 10.3390/nano13222936

**Published:** 2023-11-13

**Authors:** Radostina Kalinova, Kirilka Mladenova, Svetla Petrova, Jordan Doumanov, Ivaylo Dimitrov

**Affiliations:** 1 Institute of Polymers, Bulgarian Academy of Sciences, Academician Georgi Bonchev St., bl. 103-A, 1113 Sofia, Bulgaria; 2 Department of Biochemistry, Faculty of Biology, Sofia University “St. Kliment Ohridski”, 8 Dragan Tzankov Blvd., 1164 Sofia, Bulgaria; k_mladenova@biofac.uni-sofia.bg (K.M.); spetrova@biofac.uni-sofia.bg (S.P.); doumanov@biofac.uni-sofia.bg (J.D.)

**Keywords:** multifunctional, block copolymer, thiol-yne click, polyplex, polyion complex micelle, DNA, curcumin, drug delivery, metabolic activity

## Abstract

The synthesis of well-defined multifunctional polymers is of great importance for the development of complex materials for biomedical applications. In the current work, novel and multi-amino-functional diblock copolymer for potential gene and drug delivery applications was successfully synthesized. A highly efficient one-step and quantitative modification of an alkyne-functional polycarbonate-based precursor was performed, yielding double hydrophilic block copolymer with densely grafted primary amine side groups. The obtained positively charged block copolymer co-associated with DNA, forming stable and biocompatible nanosized polyplexes. Furthermore, polyion complex (PIC) micelles with tunable surface charge and decorated with cell targeting moieties were obtained as a result of direct mixing in aqueous media of the multi-amino-functional block copolymer and a previously synthesized oppositely charged block copolymer bearing disaccharide end-group. The obtained well-defined nanosized PIC–micelles were loaded with the hydrophobic drug curcumin. Both types of nanoaggregates (polyplexes and PIC–micelles) were physico-chemically characterized. Moreover, initial in vitro evaluations were performed to assess the nanocarriers’ potential for biomedical applications.

## 1. Introduction

Highly functionalized polymers have been extensively investigated as potential materials for various applications in electronic devices [1], fuel cells [2], and biomedicine [3]. For the preparation of multifunctional polymers tailored for the desired application, it is favorable if the respective functionalities can be added to the preformed polymer backbone in a simple and highly efficient manner under mild conditions. The specific class of reactions known as “click chemistry”, a term introduced back in 2001 by Sharpless et al., has proven to be highly effective in polymer functionalization and surface modification [4]. The “click reactions” are characterized by high yield, high selectivity, undemanding reaction conditions, and easy purification from the byproducts. They have been used for the introduction of various side-chain functionalities in linear and branched polymers [5,6]. The most popular “click reaction” is the copper-catalyzed 1,3-cycloaddition between azides and alkynes (CuCAAC) independently developed by Sharpless et al. [7] and Meldal et al. [8]. However, considering the potential biomedical applications of the modified polymer materials, a number of metal-free reactions satisfying the requirements of “click chemistry” have also been exploited [9]. These include the strain-promoted azide-alkyne cycloaddition [10], Diels–Alder [11], and thiol-based reactions [12,13]. Among them, the addition of thiols to alkynes, known as the thiol-yne “click reaction”, is of particular interest for the preparation of multifunctional materials since it allows the addition of two functional thiols to one alkyne with high efficiency under mild conditions and does not require using a significant excess of either reagent [14]. Thiol-yne “click chemistry” has been successfully applied for the synthesis of liner, graft, dendritic, and hyperbranched polymer structures, as well as for the preparation of multifunctional polymer materials via post-polymerization modifications [15,16,17,18].

Among the various multifunctional macromolecular architectures, those containing polycationic segments with high density of positive charges are particularly significant because of their capability to condense negatively charged (bio)macromolecules or low-molar-mass active substances via strong cooperative electrostatic interactions, forming nanosized aggregates for gene and drug delivery applications [19,20]. The formation of narrowly dispersed and nanosized polyion complex (PIC) micelles via simply mixing two oppositely charged doble hydrophilic block copolymers in aqueous media was first reported by Harada and Kataoka back in 1995 [21]. This alternative to the classical polymer micelles that are formed from amphiphilic block copolymers is attractive for biomedical applications since the PIC micelles are obtained in organic-solvent-free media through electrostatically driven self-association under constant thermodynamic equilibrium conditions [22]. Thus, the anionic nucleic acids (DNA or RNA) have been successfully complexed with various types of cationic polymers into, in most cases, spherical and positively charged nanoaggregates referred to as polyplexes and evaluated as non-viral and safe gene delivery vehicles [23]. However, these polyplexes formed from unmodified polycationic homopolymers are prone to interactions with blood components and tend to accumulate in tissues non-specifically. Consequently, different strategies were applied including the use of multifunctional double hydrophilic or amphiphilic positively charged copolymers in order to impart targeting, stimuli responsive, and shielding properties to the polyplexes, which are essential for increasing their stability and specific tumor accumulation [24,25,26]. PIC–micelles have also been evaluated as nanocarriers of proteins [27,28]. However, it should be noted that, in contrast to the highly charged nucleic acids, proteins possess not only charged but also neutral segments, and hydrophobic functionalities in their structures that might affect the complexation process. Finally, PIC–micelles have been used to accommodate various low-molar mass hydrophilic drugs which, in most cases, co-associate in aqueous media with the double hydrophilic oppositely charged copolymer(s) into nanosized drug delivery vehicles [29,30,31]. On the other hand, the reports concerning the evaluation of PIC micelles as nanovehicles for hydrophobic active compounds are still limited [32,33,34]. In the majority of cases, one of the pairs of copolymers involved in the electrostatically-driven association besides the charged groups possesses hydrophobic segments in order to accommodate the drug via hydrophobic interactions. The successful encapsulation of a hydrophobic bioactive substance into PIC micelles obtained in aqueous media from two oppositely charged double hydrophilic block copolymers was only reported recently [35].

The current work deals with the highly efficient and solvent-free modification of previously synthesized alkyne-multifunctional copolymer [36], thus converting it from amphiphilic to double hydrophilic block copolymer with densely grafted amine side groups. The obtained multi-amino-functionalized copolymer was then used for the formation of different nanoaggregates via electrostatic interactions with oppositely charged macromolecules. Initially, nanosized polyplexes with DNA were successfully formed. Additionally, PIC micelles with tuneable surface charge were formed simply by mixing aqueous solutions of the multi-amino-functional block copolymer and an oppositely charged double hydrophilic block copolymer. Furthermore, the obtained micelles were loaded with the hydrophobic bioactive substance curcumin. Both types of nanoaggregates intended for biomedical applications were physico-chemically characterized. Finally, initial in vitro evaluations were carried out and discussed.

## 2. Materials and Methods

### 2.1. Materials and Reagents

The chemicals were acquired from Sigma-Aldrich (St. Louis, MI, USA) unless otherwise specified. α,α’-Azoisobutyronitrile (AIBN, 98%) was purified through recrystallization from methyl alcohol. 2-Aminoethanethiol hydrochloride (AET.HCl, ≥98%) was kept under reduced pressure. The salmon sperm DNA (*M*_w_~2000 bp) was dissolved in ultra-pure water (>18 MΩ) and the obtained stock solution (100 μg mL^−1^) was used for the polyplex formation. The hydrophobic dye 1,6-diphenyl-1,3,5-hexatriene (DPH, 98%), ethidium bromide (EtBr, for molecular biology ≥95%), 2-propanol (≥99.8%), and curcumin (Curc) were used as received.

The amphiphilic block copolymer bearing alkyne side groups on each carbonate repeating unit (MPEG-*b*-PC) was synthesized under green conditions according to a previously described procedure [36]. Briefly, a methoxy-poly(ethylene glycol) (MPEG) was used as a macroinitiator for the metal-free bulk ring-opening polymerization of an alkyne-functional cyclic carbonate which proceeded at a mildly elevated temperature. ^1^H NMR (600 MHz, DMSO-d_6_, *δ*, ppm): 4.73 (s, OC*H*_2_C≡CH), 4.21–4.26 (m, OCH_2_C*H*_2_O(C=O) + OC(O)OC*H*_2_), 3.50 (s, OC*H*_2_C*H*_2_O), 3.23 (s, C*H*_3_O), 2.52 (s, CH_2_C≡C*H*), and 1.18 (s, C*H*_3_).

The negatively charged double hydrophilic block copolymer poly(ethylene glycol)-*b*-poly(L-aspartic acid) end-functionalized with lactobionic group (LBA-PEG-*b*-PLAsp) was previously obtained using a heterobifunctional PEG as a macroinitiator for the ring opening polymerization of the *N*-carboxyanhydride of *β*-benzyl-*L*-aspartate, followed by the removal of the protecting groups and the attachment of a disaccharide targeting end-group [35]. ^1^H NMR (600 MHz, D_2_O, *δ*, ppm): 4.62–4.40 (-αC*H*-NH-), 4.26–3.30 (21*H*-LBA), 3.67 (-O-C*H*_2_-C*H*_2_-O-), 2.80–2.45 (-αCH-C*H*_2_-).

### 2.2. Synthesis of Double Hydrophilic Multi-Amine-Functional Block Copolymer MPEG-b-PCA

The MPEG-*b*-PC block copolymer (0.2 g, 0,49 mmol alkyne functions), AIBN (0.059 g, 0.36 mmol), and AET.HCl (0.39 g, 3.4 mmol) were dried under a vacuum for 1 h. Following that, the reaction was allowed to proceed under argon atmosphere at 70 °C for 24 h. The product was dispersed in 2-propanol and stirred overnight. The solvent was filtered off, the residue was dissolved in distilled water, and an ultrafiltration through a membrane with MWCO 1000 Da was applied to remove the excess of the reagent. The purified copolymer was isolated from the aqueous solution via lyophilization. Yield: 0.190 g, (84%). ^1^H NMR (600 MHz, DMSO-d_6_, *δ*, ppm): 8.17 (s, -CH_2_-N*H*_3_^+^), 4.08–4.50 (m, OCH_2_C*H*_2_O(C=O) + OC(O)OC*H*_2_ + CH-C*H*_2_-S-), 3.50 (s, OC*H*_2_C*H*_2_O + CH_2_-C*H*-CH_2_-S-), 3.24 (s, C*H*_3_O), 3.02 (m, -S-CH_2_-C*H*_2_-NH_3_^+^), 2.91 (m, -S-C*H*_2_-CH_2_-NH_3_^+^ + CH_2_-CH-C*H*_2_-S-), 1.23 (s, *CH*_3_).

### 2.3. Polyplexes Formation

Typically, 1 mL from the salmon sperm DNA (100 μg mL^−1^) solution was added to an equal volume from the aqueous block copolymer (MPEG-*b*-PCA) solutions of different concentrations at ambient temperature under stirring. Thus, polyplexes varying in molar ratio between the copolymer’s amine groups and the DNA’s phosphate groups (N/P) were obtained (N/P = 1:1, 5:1, and 10:1).

### 2.4. Ethidium Bromide (EtBr) Displacement Assay

Aqueous block copolymer solutions of different concentrations (0.5 mL) were added to equal volumes of DNA/EtBr complex solutions containing 10 μg DNA and 3.0 μg EtBr to obtain polyplexes with various N/P ratios (from 0.5:1 to 20:1). The emission intensity was measured at 600 nm (exited at 530 nm) after 3 h equilibration. The relative fluorescence intensity of the polyplexes was expressed as a percentage of that of DNA/EtBr solution. The measurements were performed in triplicate.

### 2.5. Formation of Polyion Complex (PIC) Micelles

Two separate aqueous solutions of the double hydrophilic block copolymers MPEG-*b*-PCA and LBA-PEG-*b*-PLAsp bearing positively and negatively charged side groups, respectively, were initially prepared. The PIC–micelles’ formation was performed by the dropwise addition of 5 mL from the former solution to an equal volume of the latter solution upon stirring. The co-assembly of the two copolymers takes place in aqueous media as a result of the electrostatic interactions between the oppositely charged side groups, forming polyion complex micelles. An additional amount of distilled water was added in order to tune the final PIC–micelles concentration to 1 mg mL^−1^. By varying the initial concentrations of both copolymer solutions, PIC micelles with molar ratios of 1:5, 1:1, and 5:1 between the positively and the negatively charged groups of the respective double hydrophilic block copolymers were obtained.

### 2.6. Critical Micelle Concentration (CMC) Determination

The concentration at which the pair of two double hydrophilic block copolymers start to form PIC micelles in aqueous media at molar ratios 1:5, 1:1, or 5:1 between the positively and negatively charged groups was determined by applying the dye solubilization method developed by Alexandridis et al. [37]. Briefly, different concentrations in the 0.001–1.0 mg mL^−1^ range of the pair of double hydrophilic copolymers were prepared in water, followed by the addition of 10 μL from a 0.4 mM solution of the hydrophobic dye DPH in methyl alcohol. The mixtures were equilibrated overnight in the dark and their UV/Vis spectra were taken. The absorption intensity at 356 nm characteristic of the solubilized into the micelles core dye vs. the double hydrophilic block copolymers’ concentration was plotted. The onset of the micelles’ formation was determined as the intersection point of the two plotted nonparallel straight lines.

### 2.7. Curcumin Loading Evaluation and Drug Release Studies

The hydrophobic drug curcumin was loaded into the preformed PIC micelles as a solution in ethanol (1 mg mL^−1^). Typically, 1 mL of the alcoholic drug solution was added dropwise to a 10 mL vigorously stirred aqueous micelles’ dispersion with concentration of 1 mg mL^−1^. The ethanol was removed under reduced pressure and the volume of the drug-loaded aqueous dispersion was adjusted to 10 mL. As a result, the weight ratio between the PIC micelles and the drug in the feed was 10:1.

The two important parameters—drug-loading efficiency (DLE) and drug-loading capacity (DLC)—that are used to characterize the curcumin-loaded PIC–micellar systems were assessed after the separation of the unloaded drug from the curcumin loaded dispersion via filtration (filter pores size 0.45 μm). Following that, the aqueous phase was removed through lyophilization. A predetermined amount of the obtained dry drug-loaded micelles was dissolved in acetone and subjected to UV/Vis analysis. The amount of encapsulated curcumin was determined using the previously constructed calibration curve from UV/Vis measurements of the drug dissolved in acetone (λ_max_ = 418 nm, ε = 61,882 M^−1^ cm^−1^) [38]. Consequently, the DLE and DLC values were calculated from the following equations:

DLE = (weight of the loaded curcumin/weight of curcumin in the feed) × 100%(1)

DLC = (weight of the loaded curcumin/total weight of the micelles) × 100%(2)

The in vitro release of the loaded drug from the PIC–micellar nanosystems vs. time was evaluated applying the dialysis membrane method. The drug-loaded aqueous dispersion (2 mL) was separated by a Spectra/Por dialysis membrane tubing (MWCO = 50,000 Da) from the release media, comprising 100 mL of distilled water with 1% (*v*/*v*) Tween^®^ 20. The system was kept at 37 °C and gently stirred. Samples from the release media were periodically withdrawn and analyzed by UV/Vis spectroscopy. Each time when a sample was taken, an equal volume of fresh release media was added. The released drug was quantified using the UV/Vis absorption intensity at λ_max_ = 421 nm and ε = 47,827 M^−1^ cm^−1^. The in vitro release experiments were repeated three times.

### 2.8. MTT Test

The MTT assay was performed on 3 cell lines: human carcinoma hepatocytes (HepG2), human carcinoma pneumocytes (A549), and epithelial canine noncarcinoma kidney cells (MDCK II). The cells were seeded at an initial concentration of 5 × 10^5^ cells mL^−1^ 24 h prior to treatment. The treatment with polyplexes, free curcumin, empty, or curcumin-loaded PIC–micelles was performed in cell medium DMEM with 10% fetal bovine serum (FBS) and penicillin/streptomycin (Sigma-Aldrich, St. Louis, MI, USA/Merck, Rahway, NJ, USA) for 24 h. Untreated cells were used as a control. After treatment, cells were incubated with 0.05 mg mL^−1^ MTT reagent (Sigma-Aldrich/Merck). The formazan crystals, which appeared as a result of a reaction between the MTT reagent and the dehydrogenase enzymes in the cells, were dissolved in DMSO. The absorbance of the samples was read at a wavelength of 562 nm. Metabolic activity was calculated as a percentage of absorbance of the sample relative to the absorbance of the control.

### 2.9. Characterization Methods

The proton nuclear magnetic resonance (^1^H NMR) spectroscopic analyses were performed on a Bruker Avance NEO spectrometer (Billerica, MA, USA) operating at 600 MHz frequency. A deuterated dimethyl sulfoxide (DMSO-d_6_) was used to dissolve the samples. The UV/Vis spectroscopic measurements were recorded on a Beckman Coulter (Brea, CA, USA) instrument equipped with a Peltier temperature controller. The infrared spectroscopic analyses were run on an IRAffinity-1 Fourier Transform Infrared (FTIR) spectrophotometer from Shimadzu (Kyoto, Japan). The fluorescence measurements were conducted on Agilent Cary Eclipse Fluorescence spectrophotometer (Santa Clara, CA, USA). The morphology of the various types of nanoaggregates obtained was examined using a high-resolution transmission electron microscope (TEM) operating at 200 kV (HRTEM JEOL JEM-2100, Tokyo, Japan). The images were captured using a digital camera with a charge-coupled device (CCD) image detector (GATAN Orius 832 SC1000, Pleasanton, CA, USA) and were processed with a GATAN Microscopy Suite (GMS) 3.4 Software. The size–distribution profiles and the mean sizes of the obtained nanoaggregates in aqueous dispersion were evaluated on NanoBrook Plus PALS equipment (Holtsville, NY, USA), employing dynamic light scattering (DLS) at 90°. For the nanoparticles with a spherical shape, the hydrodynamic diameter (*d_H_*) was calculated from the Stokes–Einstein equation:

*d_H_* = *kT*/(3*πηD*), (3)

(*k* is the Boltzmann’s constant, *T* is the absolute temperature (K), *η* is the solution viscosity, and *D* is diffusion coefficient).

The electrophoretic mobility of the positively or negatively charged polyion complex nanoaggregates’ dispersions was estimated using the NanoBrook Plus PALS equipment and utilizing the phase analysis light scattering (PALS). The zeta-potential (ζ, mV) as a measure of the nanoparticles’ surface charge was obtained from the Smoluchowski equation:

ζ = 4*πημ*/*ε*, (4)

(*η* is the solution viscosity; *μ* is the electrophoretic mobility, *ε* is the dielectric constant of the solvent).

Three independent DLS measurements were run on each sample. The values of the *d_H_* and the size distribution were presented as an average from the three runs. The ζ was presented as an average from twenty runs per sample.

## 3. Results and Discussion

### 3.1. Solvent-Free Synthesis of Multi-Amino-Functional Diblock Copolymer MPEG-b-PCA

**Scheme 1:**
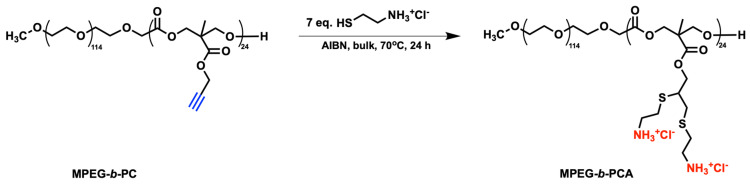
Synthetic path under solvent-free conditions to multi-amino-functional diblock copolymer MPEG-*b*-PCA.

We have recently demonstrated the green synthetic route towards a well-defined amphiphilic multi-alkyne-functional diblock copolymer comprising biocompatible and biodegradable blocks (MPEG-*b*-PC) [36]. The copolymer was successfully evaluated as a nanosized carrier of hydrophobic drugs. Herein, the green synthetic path was extended in order to modify the copolymer and to convert it to a highly amine-functionalized double hydrophilic block copolymer for potential biomedical applications. Thus, for the formation of the multifunctional polymer structure, a thermally induced thiol-yne “click” radical reaction was exploited as it appears to be a very robust and versatile method that tolerates a variety of functional groups. The attachment of two alkylamine side groups per each pendant alkyne function of the MPEG-*b*-PC block copolymer was performed according to Scheme 1.

**Figure 1:**
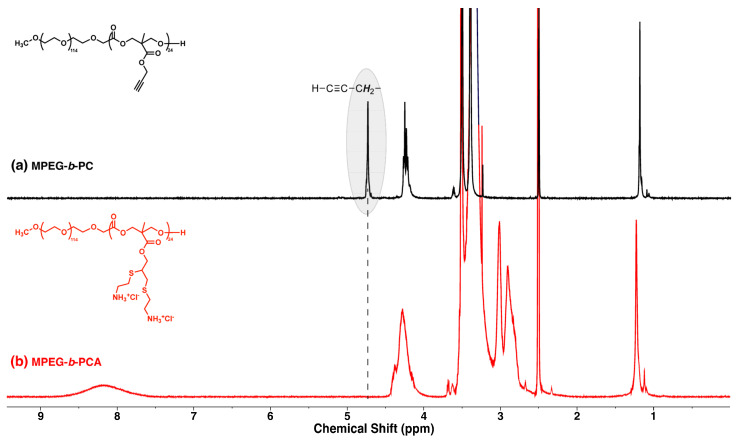
Proton NMR overlay of: (**a**) alkyne-functional precursor (MPEG-*b*-PC); and (**b**) multi-amino-functional double hydrophilic block copolymer (MPEG-*b*-PCA).

An excess of 2-aminoethanethiol hydrochloride was used as a bifunctional reagent in the presence of AIBN as a radical initiator. The modification process proceeded in bulk at relatively low temperature of 70 °C under conditions that are close to the requirements for the green synthesis. According to the well-established two-step mechanism of the thiol-yne “click” radical reaction, initially each alkyne functional group reacts with a single thiol, thus forming vinyl sulfide followed by the rapid addition of a second thiol, yielding the final 1,2-disubstituted product [39]. After the reaction’s completion, the product was thoroughly purified—initially via extraction with 2-propanol followed by ultrafiltration in water in order to remove the initiator and excess of AET.HCl. The completion of the modification reaction was evidenced by the ^1^H NMR analyses of both copolymers performed in DMSO-d_6_ (Figure 1). The proton intensity peak at 4.73 ppm characteristic for the methylene groups located next to the alkyne functionalities of the precursor MPEG-*b*-PC (Figure 1a) has completely disappeared from the spectrum of the purified final product (Figure 1b). Moreover, there are no signals corresponding to vinyl protons in the modified copolymer spectrum, indicating that the 1,2-disubstitution proceeded to completion. On the other hand, new peaks at 8.17, 3.03, and 2.91 ppm corresponding to the protons from the attached ammonium and methylene side groups are clearly visible in the ^1^H NMR spectrum of MPEG-*b*-PCA (Figure 1b). The integral intensities of these protons compared to that of the methyl protons from the polycarbonate backbone at 1.23 ppm also indicates the complete modification of each repeating unit with two alkyl amine side groups.

**Figure 2:**
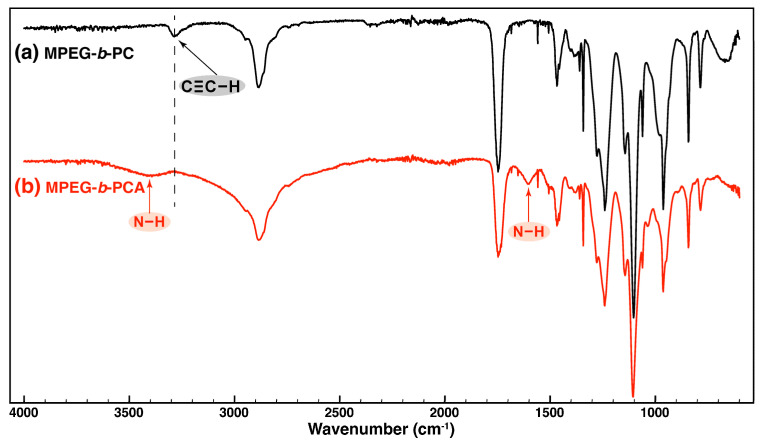
FTIR spectra of: (**a**) alkyne-functional amphiphilic block copolymer precursor (MPEG-*b*-PC); and (**b**) multi-amino-functional double hydrophilic block copolymer (MPEG-*b*-PCA).

The successful modification step was also evidenced by FTIR analyses performed on the block copolymer before and after the thiol-yne “click” reaction (Figure 2).

The stretching vibrations of the C≡C–H side groups in the precursor copolymer MPEG-*b*-PC characterized with the IR-band at 3287 cm^−1^ and shown on Figure 2a completely disappeared from the spectrum of the modified copolymer MPEG-*b*-PCA. Additional bands at 1601 cm^−1^ and 3404 cm^−1^ corresponding to N–H bending and stretching vibrations could be clearly identified in the spectrum of the double hydrophilic copolymer. They were assigned to the newly attached amine side groups onto the polycarbonate backbone (Figure 2b).

### 3.2. Formation and Evaluation of Polyplexes between the Double Hydrophilic Block Copolymer MPEG-b-PCA and DNA

**Scheme 2:**
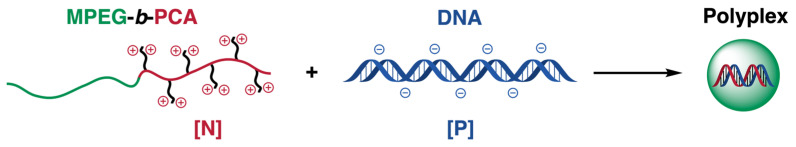
Polyplex formation between the multi-amino-functional diblock copolymer MPEG-*b*-PCA and DNA.

The successfully synthesized and characterized novel double hydrophilic block copolymer densely functionalized with primary amine side groups is a promising candidate for condensation of DNA into nanosized polyplexes via electrostatic interactions and potential gene delivery applications (Scheme 2). 

**Figure 3:**
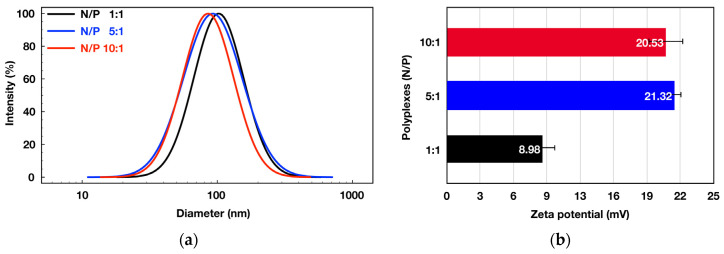
Size distribution curves obtained by DLS (**a**), and zeta potentials obtained by PALS (**b**) of polyplexes prepared in aqueous media at different N/P ratios: 1:1 (d = 122.6 ± 0.18 nm, PdI: 0.246, ζ = 8.98 ± 1.19 mV), 5:1 (d = 93.7 ± 5.72 nm, PdI: 0.267, ζ = 21.32 ± 0.66 mV), 10:1 (d = 85.9 ± 1.88 nm, PdI: 0.233, ζ = 20.53 ± 1.66 mV).

Consequently, aqueous solutions of DNA fixed at 100 μg mL^−1^ and different concentrations of the multifunctional diblock copolymer MPEG-*b*-PCA were prepared separately and mixed. The electrostatically driven co-assembly of the oppositely charged macromolecules resulted in the formation of polyplexes with three different N/P molar ratios. These include one polyplex formed under equimolar conditions (N/P = 1:1) and two polyplexes with a molar excess of primary amine groups (N/P = 5:1 and N/P = 10:1). The obtained polyplexes were subjected to DLS analyses. The formed polyplexes are with average diameters in the 122–86 nm range with relatively narrow size distributions (Figure 3a). 

A clear decrease in the nanoparticles’ size is observed with the increase of the N/P molar ratio from 1:1 to 5:1. However, a further increase of N/P ratio to 10:1 does not lead to significant changes in polyplexes’ average size. Therefore, it might be concluded that the optimal molar ratio between the oppositely charged groups of the two macromolecules for the nanosized polyplexes formation is 5:1. The zeta potential measurements of the different polyplexes revealed the tendency of increasing of the positive surface charge with the increase of N/P ratio (Figure 3b). Thus, the polyplexes prepared at N/P 1:1 exhibit slightly positive, close to neutral surface charge, whereas those prepared at N/P 5:1 and 10:1 possess strongly positive surface charge of about 20 mV. As with the size distribution measurements, there is no detectable change in the particles’ surface charge when the N/P ratio was increased from 5:1 to 10:1.

The binding ability of the multi-amino-functional block copolymer to DNA is essential for the efficient protection of the nucleic acid against degradation by nucleases during the transportation to the target cells. It was evaluated by ethidium bromide displacement assay. It is well-known that ethidium bromide forms intercalating complexes with double helical nucleic acids resulting in a significant enhancement of their fluorescent intensity. This enhanced intensity is quenched upon the DNA condensation with the cationic groups of the added block copolymer solution. As shown in Figure S1, the addition of increasing concentrations of the block copolymer to the DNA/EtBr solutions resulted in a gradual decrease of fluorescent intensity, which was completely quenched above N/P ratio of 2.5. The 100% quenching effect indicates the strong ability of the double hydrophilic block copolymer to bind DNA.

The multifunctional block-copolymer’s capacity to release DNA in the endosomes’ acidic environment was evaluated using an acidic buffer (pH 5.5) in which the freshly prepared at N/P 5:1 ratio aqueous polyplex dispersion was incubated. The changes in particles’ average diameter were monitored by DLS measurements. Upon the addition of the buffer solution, a significant increase in the particles average diameter was instantly detected. After 30 min of incubation in acidic conditions, only large micrometer-sized particles with a broad size distribution were detected and were attributed to the released from the polyplex DNA (Figure S2). The obtained initial results indicate that the newly synthesized multi-amino-functional block copolymer would be capable of successfully releasing the DNA into the endosomes’ acidic environment.

**Figure 4:**
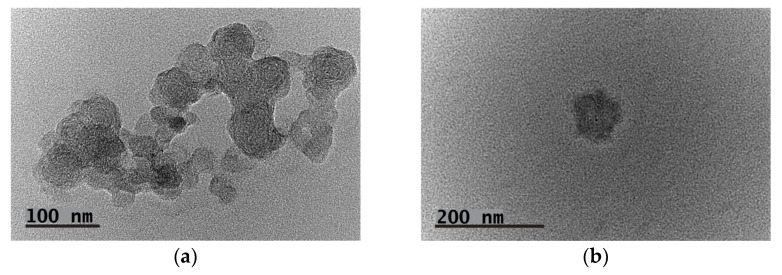
TEM micrographs of: (**a**) cluster of polyplexes (d = 63.82 ± 6.33 nm); (**b**) individual nanoparticle (d = 79.88 nm), obtained at N/P 5:1.

Transmission electron microscopy was used to study the morphology of the polyplexes (Figure 4). The TEM images show the presence of aggregates from the nanosized polyplexes (Figure 4a). Their spherical shape can be clearly seen from the TEM micrograph of a single nanoparticle (Figure 4b).

The measured average diameters of the polyplexes are somewhat smaller than those obtained from the DLS analyses due to the specific conditions for the sample preparation required by the two techniques. The DLS measurements were performed on fully hydrated nanoparticles, whereas the smaller-sized polyplexes visualized by TEM resulted from the solvent removal on the grid during the specimen preparation.

**Figure 5:**
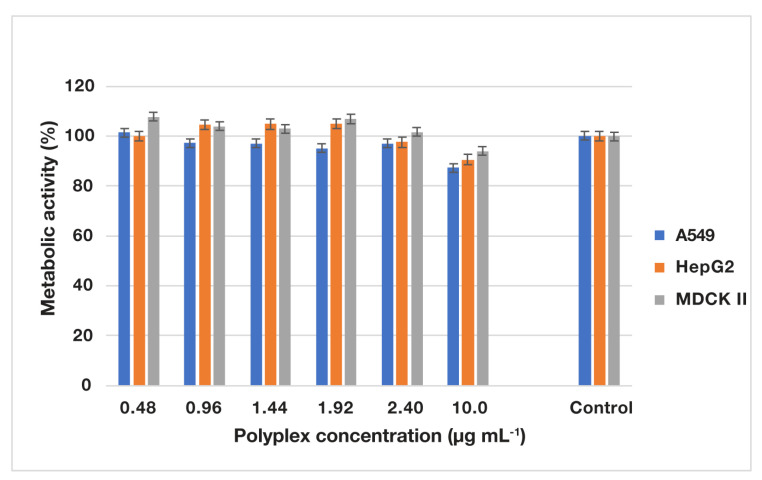
Metabolic activity of A549, HepG2, and MDCK II cells (via MTT test) after 24 h of incubation with different polyplex (N/P 5:1) concentrations. The results are presented as % of untreated controls and are expressed as an arithmetic mean of three independent experiments ± SE.

Finally, in vitro analyses on metabolic activity of panel cell lines treated with different polyplex concentrations were performed to evaluate the potential of the newly obtained nanoaggregates as safe DNA delivery systems. The MTT analyses were run on normal (MDCK II) and two cancer (A549 and HepG2) cell lines treated with increasing polyplex concentrations. The results are presented in Figure 5. The results showed that the polyplex (N/P 5:1) used in the concentration range from 0 to 10 μg mL^−1^ caused only negligible antiproliferative effects on the three cell lines of different origin. The estimated reduced level of metabolic activity in the various cell lines was in the 6–12% range compared to the corresponding controls at the highest polyplex concentration studied. The established lack of cytotoxic effect of the polyplexes is a prerequisite for their potential application as DNA nanocarriers.

### 3.3. Polyion Complex (PIC) Micelles Formation between the MPEG-b-PCA and LBA-PEG-b-PLAsp Functional Synthetic Block Copolymers Bearing Polycationic and Polyanionic Segments

**Scheme 3:**
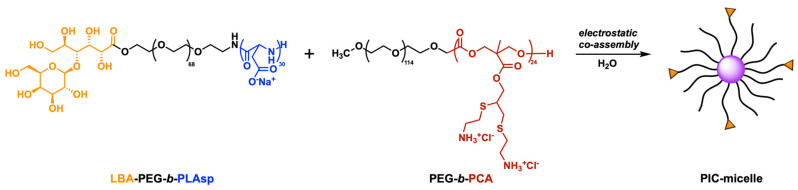
PIC–micelles formation between the multi-amino-functional block copolymer MPEG-*b*-PCA and poly(ethylene glycol)-*b*-poly(L-aspartic acid) block copolymer functionalized with lactobionic end-group (LBA-PEG-*b*-PLAsp).

In addition to the polyplex formation leading to DNA or RNA condensation, the electrostatic interactions between rationally designed functional oppositely charged synthetic block copolymers attract considerable attention due to the numerous possibilities towards multifunctional nanosized PIC–micelles for hydrophilic or charged drug delivery applications. Their potential application as nanocarriers was recently extended to hydrophobic drugs [35]. Thus, the possibility of PIC–micelles formation between the newly synthesized multi-amino-functional block copolymer MPEG-*b*-PCA and previously synthesized poly(ethylene glycol)-*b*-poly(L-aspartic acid) block copolymer end-functionalized with lactobionic targeting end-group (LBA-PEG-*b*-PLAsp) was further exploited. Since both copolymers are water soluble, the PIC–micelles formation was performed in aqueous media by simply mixing the pair of oppositely charged macromolecules as shown in Scheme 3. The molar ratio between the corresponding amine and carboxylic side groups of the two block copolymers varied from 1:5 to 5:1 in order to obtain three types of PIC–micellar dispersions (PIC 1:5, PIC 1:1, PIC 5:1). For all of them, the final concentration was tuned to 1 mg mL^−1^.

#### 3.3.1. Physico-Chemical Characterization

In order to estimate the thermodynamic stability of the aggregates the critical micelle concentration (CMC), i.e., the concentration at which the micelles start to form, was evaluated. The spectroscopic method reported by Alexandridis et al. was applied [37]. A hydrophobic dye (DPH) was injected as a methanolic solution to a series of aqueous solutions of MPEG-*b*-PCA and LBA-PEG-*b*-PLAsp with increasing concentrations. Once solubilized in the polyelectrolyte complex interior of the forming micelles, the dye can be detected by UV/Vis spectroscopy. The intensity of dye’s UV-absorption increases with the micelles’ concentration. The CMC values for the three types of PIC micelles were estimated from the constructed absorbance intensity vs. concentration graphs (Figure S3). The obtained CMC values were between 0.016 and 0.041 mg mL^−1^. All of them are in the micromolar range (0.75–1.06 μM), pointing to a decent thermodynamic stability of the micellar systems at low concentrations and their potential to be used for hydrophobic drug delivery.

**Figure 6:**
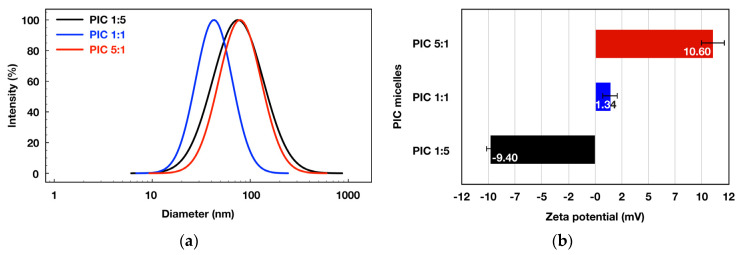
Size distribution curves obtained by DLS (**a**), and zeta potentials obtained by PALS (**b**) of PIC–micelles’ dispersions prepared at different molar ratios between the copolymers’ oppositely charged side groups: PIC 1:5 (d = 74.21 ± 1.47 nm, PdI: 0.416, ζ = –9.40 ± 0.36 mV), PIC 1:1 (d = 41.04 ± 1.53 nm, PdI: 0.223, ζ = 1.34 ± 0.66 mV), PIC 5:1 (d = 78.52 ± 2.24 nm, PdI: 0.282, ζ = 10.60 ± 1.09 mV).

The aqueous PIC–micelles dispersions were further characterized by DLS and PALS measurements (Figure 6).

The measurements were run on dispersions (1 mg mL^−1^) with concentrations that are significantly above the estimated CMC values. The results showed that all three types of dispersions comprise nanosized particles (Figure 6a). The average PIC–micelles’ diameters were between 79 and 41 nm. It should be noted that in the cases of PIC–micelles prepared with an excess of negatively or positively charged block copolymers (PIC 1:5 and PIC 5:1), the average diameters were around 75–79 nm with rather broad size distributions. On the contrary, the PIC–micelles formed using an equimolar ratio between the positively and the negatively charged groups are characterized with much smaller average hydrodynamic diameter of 41 nm and much narrower size distribution (Figure 6a).

The results from zeta potential measurements of the PIC–micelles presented on Figure 6b demonstrate that the PIC–micelles’ surface charge can be easily controlled by varying the ratio between the oppositely charged groups of the pair block copolymers. Thus, the zeta-potential of the PIC micelles prepared at molar ratio 1:1 is close to zero, whereas those of PIC 1:5 and PIC 5:1 micelles prepared with an excess from one of the charged copolymers are −9.40 and +10.60, respectively. However, it is worth noting that the changes in the zeta potentials with varying the molar ratio of the charged groups are much less pronounced for the PIC micelles as compared to polyplexes (Figure 3b). This could be attributed to the comparable molar masses and number of negatively and positively charged groups in the double hydrophilic block copolymers forming the PIC micelles. Additionally, the shielding effect of both hydrophilic polyether blocks is much more effective for the PIC micelles.

**Figure 7:**
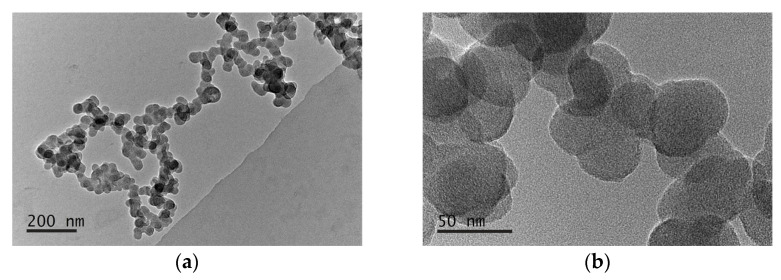
TEM micrographs of PIC 1:1 micelles prepared at equimolar ratio between the oppositely charged groups (d = 38.55 ± 3.31 nm): (**a**) low (×20,000) magnification; (**b**) high (×100,000) magnification.

The PIC–micelles’ morphology (shape and size) was visualized by TEM analyses (Figure 7). 

The low-magnification TEM image revealed an aggregated chain-like cluster of spherical nanoparticles with uniform sizes (Figure 7a). The mostly spherical shape of the PIC micelles can be verified more clearly from the high-magnification TEM image presented on Figure 7b. The measured average particles’ diameters of 38.5 nm are slightly smaller but still very close to those obtained from the DLS analyses. The slight deviations in the size and shape of the PIC–micelles could be attributed to sample preparation method for TEM analysis.

As a result of PIC–micelles physico-chemical characterization, it might be concluded that the PIC micelles formed at equimolar charged groups’ ratio (PIC 1:1) are the most suitable candidates for potential biomedical applications considering the CMC value, optimal size, and size distribution, as well as their close to neutral surface charge that would provide a “stealth” effect during the blood circulation. The particles’ hydrophilic and neutral PEG-shell formed from both copolymers is well-known for its antifouling and “stealth” properties that inhibit blood protein adsorption [40]. However, there is a risk of reduced cellular uptake which is characteristic for the PEG-coated drug nanocarriers (“PEG dilemma”) [41]. That issue was addressed by the presence of cell targeting lactobionic ligands on the PIC–micelles surface.

#### 3.3.2. Curcumin Loading Evaluation and In Vitro Drug Release Studies

The loading experiments were performed with curcumin (Curc), a well-known polyphenolic natural substance exhibiting a plethora of biological activities [42]. The inherent drug’s hydrophobic nature, low stability, and poor bioavailability could be overcome via its encapsulation in various nanoformulations [43,44]. Thus, the obtained and characterized PIC micelles were evaluated as potential Curc nanocarriers. The hydrophobic drug was introduced as an ethanolic solution to stirred aqueous dispersions of the PIC micelles varying in the surface charge. After ethanol evaporation, the concentration of the drug-loaded dispersions was tuned to 1 mg mL^−1^ with a ratio between the micelles and Curc 10:1 (*wt*/*wt*). The unloaded drug was removed through a membrane syringe filtration and the curcumin-loaded PIC micelles were further subjected to analyses. The drug loading efficiency (DLE) was estimated after the aqueous dispersions lyophilization followed by dissolving of a predetermined amount of the residue into acetone. The amount of the encapsulated curcumin was determined from the UV-spectrum of the acetone solution with known concentration. The values of DLE for the different PIC micelles were calculated according to Equation (1) and were in the 56–76% range. The highest DLE was estimated for the PIC micelles formed at equimolar charged groups’ ratio (PIC 1:1) characterized with the most compact structure. It might be speculated that due to the optimal composition of these aggregates the drug penetrates more easily through the micelle’s shell to reach the polyelectrolyte complex core. According to Equation (2), the calculated drug loading capacity (DLC) values for PIC 1:5, PIC 1:1, and PIC 5:1 micelles were 7.3, 6.9, and 5.1, respectively.

The curcumin-loaded PIC micelles were also subjected to DLS and PALS analyses. A slight but clearly noticeable increase of about 5–8 nm in the average particles diameters was recorded after the Curc incorporation into the micelles (Figure S4a). The increase could be attributed to the polyelectrolyte complex interior swelling due to the drug solubilization. Interestingly, in line with our previously obtained results for other types of PIC micelles [35], the drug-loaded nanocarriers exhibit extremely narrow size distribution (between 0.06 and 0.09) as compared to their empty analogues, for which PdIs vary in a wide range (Table 1). This is likely due to some polymer chains rearrangements taking place as a result of the addition of an ethanol to the aqueous dispersions and its subsequent removal under vacuum combined with the process of the drug solubilization into the preformed PIC micelles. The zeta potential measurements on the drug-loaded PIC micelles revealed that practically there is no change in the particles’ surface charge after the Curc encapsulation (Figure S4b). These results suggest that the encapsulated drug is located mainly into the polyelectrolyte complex core and in the interior of the shell, away from the surface. The characteristics of the non-loaded and the drug-loaded PIC–micelles are listed in Table 1.

**Figure 8:**
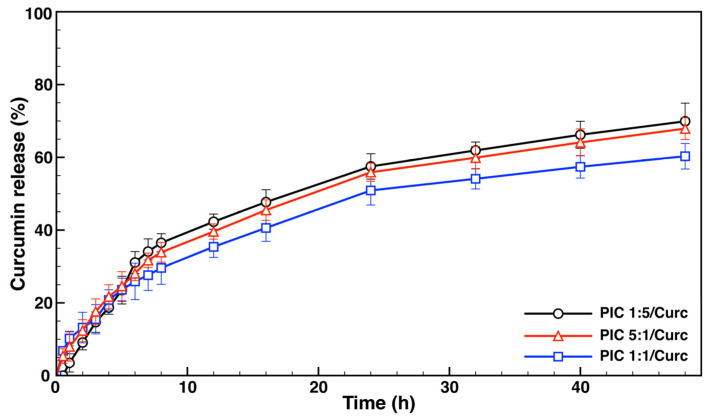
In vitro release profiles of curcumin from the PIC micelles formed at different molar ratios between the oppositely charged groups of the double hydrophilic block copolymers MPEG-*b*-PCA and LBA-PEG-*b*-PLAsp in distilled water (+1% (*v*/*v*) Tween^®^ 20). The data are expressed as mean value ± SD, n = 3.

In vitro drug release profiles were assessed for the curcumin-loaded PIC micelles. The aqueous micellar dispersions were placed in dialysis tubing with MWCO 50,000 Da and were immersed into the aqueous release media containing 1% (*v*/*v*) Tween^®^ 20 as a drug solubilizing agent. The system was kept at 37 °C under gentle stirring. Periodically, samples from the release media were withdrawn and analyzed by UV/Vis spectroscopy. The overall volume was maintained constant via the addition of fresh amounts of the release media after each sample withdrawal. The cumulative curcumin release vs. time profiles for the three drug-loaded PIC–micellar systems are presented in Figure 8. The release profiles studied are similar and biphasic for the three systems and are characterized by an initial burst drug release during the first 8 h followed by a sustained release. The first-order-like kinetics of the initial stage might be due to the release of curcumin (30–36%) that is located in the vicinity of the polyelectrolyte complex core. The following stage represents a much slower and sustained curcumin release, which could be attributed to the release of drug located into the polyelectrolyte complex core. The drug release at this stage is most likely driven by the gradual core expansion. The obtained release profiles clearly indicate that the initial burst effect is less pronounced in the case of the PIC–micelles formed at equimolar ratio between the oppositely charged block copolymers’ side groups as compared to the positively and negatively surface charged micelles. Moreover, after 48 h, 60% of loaded curcumin was released from the PIC 1:1/Curc micelles, whereas after the same period of time 70 and 68% curcumin was released from the charged micelles.

The results from drug loading and release evaluations indicate that the PIC 1:1 micelles are characterized by the highest drug loading efficiency and most likely would be able to preserve the cargo to a higher extent during blood circulation. However, more in vitro and in vivo evaluations are needed to prove this assumption.

#### 3.3.3. In Vitro Metabolic Activity Assessment of Various Cells Treated with Empty and Drug-Loaded PIC Micelles

**Figure 9:**
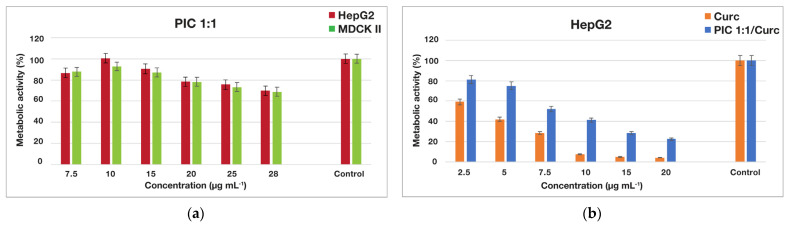
Metabolic activity via the MTT test after 24 h of incubation of: (**a**) HepG2 and MDCK II cells treated with different PIC–micelles concentrations; (**b**) HepG2 cells treated with different free (Curc) and micellar (PIC 1:1/Curc) curcumin concentrations. The results are presented as % of untreated controls and are expressed as an arithmetic mean of three independent experiments ± SE.

The MTT tests were performed with PIC micelles formed at an equimolar ratio between the charged groups of the constituent block copolymers (PIC 1:1), as they were identified as the most promising candidates for biomedical applications. Initially, the potential application of the newly prepared PIC micelles as safe drug nanocarriers was evaluated on normal (MDCK II) and cancer (HepG2) cell lines. The concentration-dependent metabolic activity of both cell lines treated with the empty PIC–micelles dispersion is presented in Figure 9a. The micellar nanocarriers caused low antiproliferative effects on the two cell lines of different origin since they failed to induce 50% inhibition of metabolic activity in the whole studied concentration interval. At the highest PIC–micelles’ concentration applied the reduced level of metabolic activity was estimated to be 30% versus the untreated controls for both cell lines. The obtained results indicated that the PIC micelles can be further evaluated as safe drug nanocarriers. The next step was to evaluate the in vitro metabolic activity of cancerous HepG2 cells treated with increasing concentrations of curcumin-loaded PIC–micelles (PIC 1:1/Curc) in comparison with the metabolic activity of cells treated with the same concentrations of free curcumin (introduced as an ethanolic solution). The results clearly demonstrated that the drug-loaded PIC micelles significantly inhibit the metabolic activity of the cancer cell line (Figure 9b). As the nanocarrier exhibits low antiproliferative effect in the whole concentration range (Figure 9a), the observed effect is due mainly to the released curcumin. The free drug demonstrates a more pronounced inhibition effect in the same concentration range as compared to the micellar curcumin.

This result can be explained by the fact that the micellar drug needs more time to be released from the nanocarrier and to reveal its biological activity. Moreover, free curcumin was introduced into the media as an ethanol solution. Therefore, it is expected that in future in vivo evaluations the drug-loaded PIC–micellar nanocarriers will fully reveal their advantages.

## 4. Conclusions

A polycarbonate-based multi-alkyne functional amphiphilic block copolymer precursor was successfully converted into a double hydrophilic block copolymer under green synthetic conditions. A highly efficient and solvent-free thiol-yne “click” reaction was applied to quantitatively attach two alkylammonium chloride side groups per each carbonate repeating unit of the block copolymer. The obtained multi-amino-functional block copolymer was used to condense DNA into biocompatible nanosized polyplexes for potential gene delivery applications. The newly synthesized block copolymer was further used to form polyion complex (PIC) micelles through electrostatic interactions with an oppositely charged poly(L-aspartate)-based block copolymer. By varying the molar ratio between the two copolymers, nanosized PIC–micelles of different surface charge were obtained and characterized. The micelles were loaded with the hydrophobic drug curcumin and the most promising candidate for drug delivery applications was identified after a series of physico-chemical and in vitro biological evaluations.

## Figures and Tables

**Table 1:** Characteristics of various types of PIC micelles before and after the curcumin loading.

Non-Loaded PIC Micelles				Curcumin-Loaded PIC Micelles					
Code	CMC ^a^ (mg mL^−1^)	d ^b^ (nm)	PdI ^b^	ζ ^b^ (mV)	d ^b^ (nm)	PdI ^b^	ζ ^b^ (%)	DLE ^c^ (%)	DLC ^c^ (%)
PIC 1:5	0.041	74.21 ± 1.47	0.416	−9.40 ± 0.36	79.43 ± 0.69	0.060	−8.65 ± 0.56	66	7.3
PIC 1:1	0.016	41.04 ± 1.53	0.223	1.34 ± 0.66	47.81 ± 1.20	0.088	1.96 ± 0.85	76	6.9
PIC 5:1	0.034	78.52 ± 2.24	0.282	10.60 ± 1.09	86.62 ± 1.57	0.091	12.10 ± 1.29	56	5.1

^a^ Critical micelle concentration (CMC) estimated by UV/Vis spectroscopy. ^b^ Average hydrodynamic diameters (d), size distributions (PdI), and zeta potentials (ζ) assessed from DLS and PALS analyses. ^c^ Drug-loading efficiency (DLE) and drug-loading capacity (DLC) estimated by UV/Vis spectroscopy.

## Data Availability

Data are contained within the article and supplementary materials.

## Supplementary Materials

The following supporting information can be downloaded at: https://www.mdpi.com/article/10.3390/nano13222936/s1, Figure S1: Ethidium bromide assay for polyplexes prepared at different N/P ratios. The data are expressed as mean value ±SD, *n* = 3; Figure S2: Size distribution curves obtained by DLS: (a) of aqueous polyplex dispersion prepared at N/P ratio 5:1 (d = 86.12 ± 0.60 nm, PdI: 0.185); and (b) after 30 min of incubation into acidic buffer at pH 5.5 (d = 1059.68 ± 0.02 nm, PdI: 0.519); Figure S3: The effect of the oppositely charged block copolymers concentration on the absorption intensity of DPH at 356 nm in aqueous media for PIC micelles formation at: (a) 1:5, (b) 1:1, and (c) 5:1 ionic groups’ molar ratio; Figure S4: Size distribution curves obtained by DLS (a), and zeta potentials obtained by PALS (b) of curcumin-loaded PIC-micelles’ dispersions prepared at different molar ratios between the copolymers’ oppositely charged side groups: PIC 1:5/Curc (d = 79.43 ± 0.69 nm, PdI: 0.060, ζ = –8.65 ± 0.56 mV), PIC 1:1/Curc (d = 47.81 ± 1.20 nm, PdI: 0.088, ζ = 1.96 ± 0.85 mV), PIC 5:1/Curc (d = 86.62 ± 1.57 nm, PdI: 0.091, ζ = 12.10 ± 1.29 mV).
