# Supplementary material for: Solvent-Free Synthesis of Multifunctional Block Copolymer and Formation of DNA and Drug Nanocarriers
Source: Nanomaterials (Basel). 2023 Nov 13;13(22):2936. doi: 10.3390/nano13222936 (PMC10675335; doi:10.3390/nano13222936)
Supplement: Supplementary file 1 [file nanomaterials-13-02936-s001.zip › nanomaterials-2686811-supplementary.pdf]

Supplementary Materials

Solvent-free Synthesis of Multifunctional Block Copolymer and Formation of DNA and Drug Nanocarriers

Radostina Kalinova [1,*], Kirilka Mladenova [2], Svetla Petrova [2], Jordan Doumanov [2] and Ivaylo Dimitrov [1,*]

[1] *Institute of Polymers, Bulgarian Academy of Sciences, Akad. Georgi Bonchev St., bl. 103-A, 1113 Sofia, Bulgaria;*

[2] *Faculty of Biology, Sofia University "St. Kliment Ohridski", 8 Dragan Tzankov Blvd., 1164 Sofia, Bulgaria*

* Correspondence: kalinova@polymer.bas.bg (R.K.); dimitrov@polymer.bas.bg (I.D.)

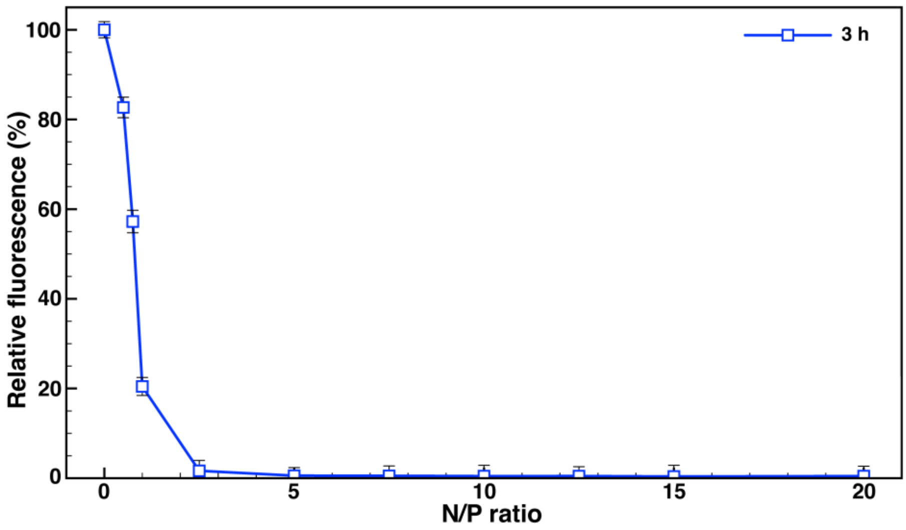

Figure S1. Ethidium bromide assay for polyplexes prepared at different N/P ratios. The data are expressed as mean value ±SD, n = 3.

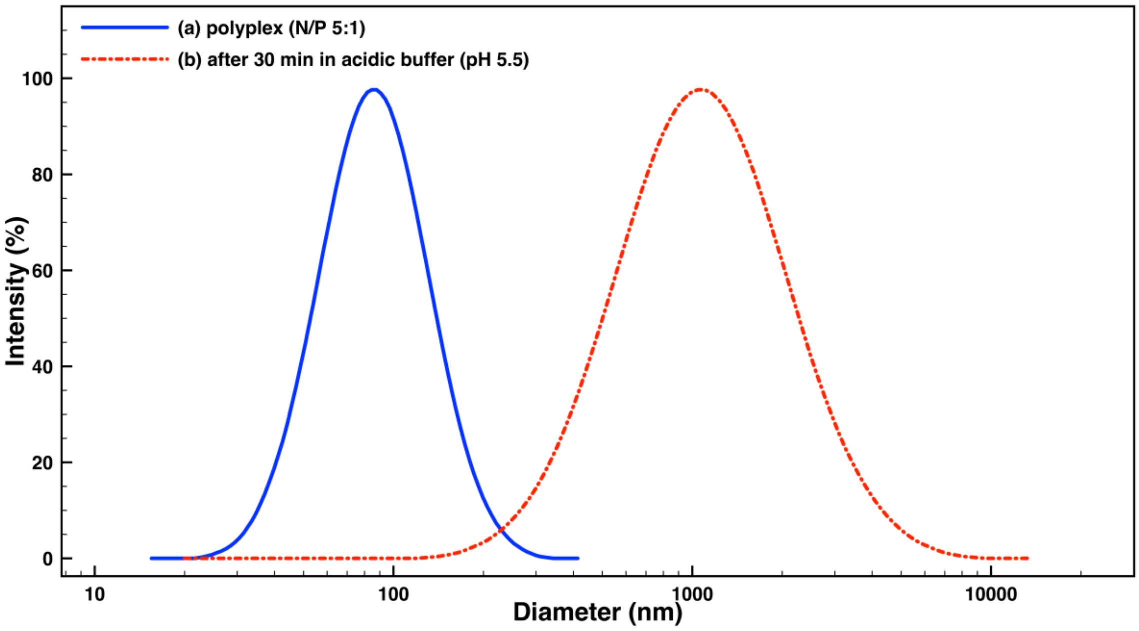

Figure S2. Size distribution curves obtained by DLS: (**a**) of aqueous polyplex dispersion prepared at N/P ratio 5:1 (d = 86.12 ± 0.60 nm, PdI: 0.185); and (**b**) after 30 min of incubation into acidic buffer at pH 5.5 (d = 1059.68 ± 0.02 nm, PdI: 0.519).

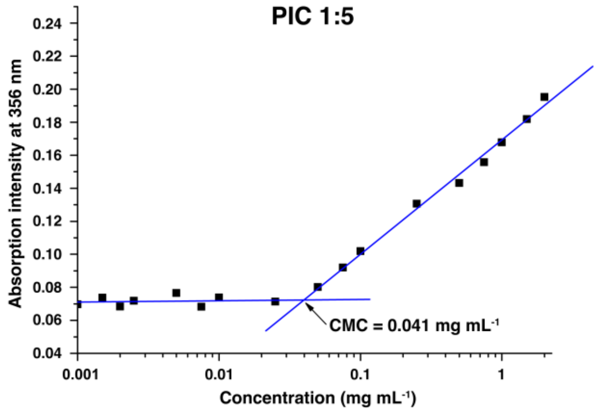

(**a**)

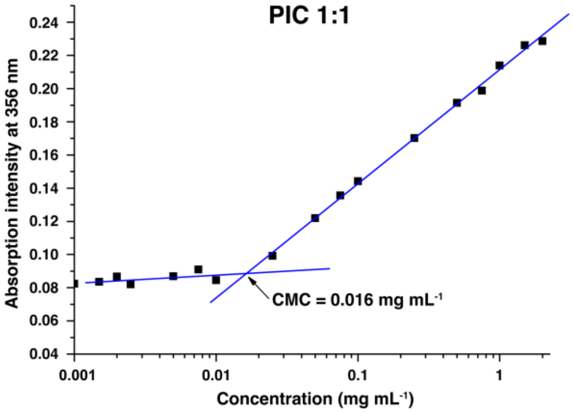

(**b**)

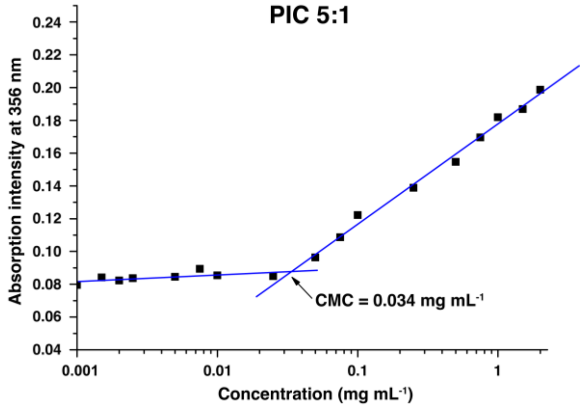

(**c**)

Figure S3. The effect of the oppositely charged block copolymers concentration on the absorption intensity of DPH at 356 nm in aqueous media for PIC micelles formation at: (**a**) 1:5, (**b**) 1:1, and (**c**) 5:1 ionic groups' molar ratio.

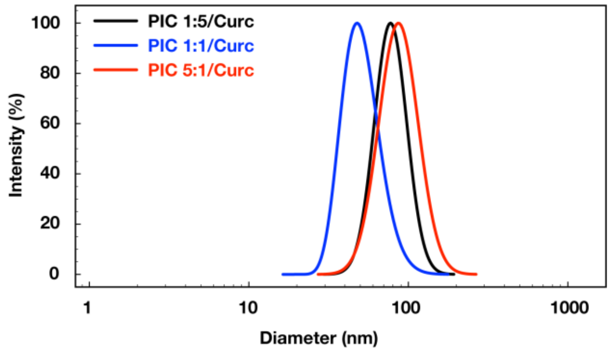

(**a**)

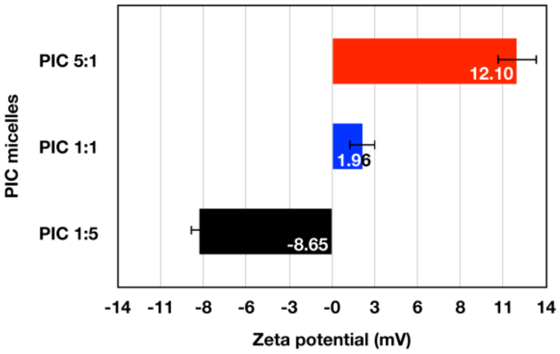

(**b**)

Figure S4. Size distribution curves obtained by DLS (**a**), and zeta potentials obtained by PALS (**b**) of curcumin-loaded PIC-micelles' dispersions prepared at different molar ratios between the copolymers' oppositely charged side groups: PIC 1:5/Curc (d = 79.43 ± 0.69 nm, PdI: 0.060, ζ = –8.65 ± 0.56 mV), PIC 1:1/Curc (d = 47.81 ± 1.20 nm, PdI: 0.088, ζ = 1.96 ± 0.85 mV), PIC 5:1/Curc (d = 86.62 ± 1.57 nm, PdI: 0.091, ζ = 12.10 ± 1.29 mV).
